# Magnetic core–shell nanowires as MRI contrast agents for cell tracking

**DOI:** 10.1186/s12951-020-00597-3

**Published:** 2020-03-12

**Authors:** Aldo Isaac Martínez-Banderas, Antonio Aires, Sandra Plaza-García, Lorena Colás, Julián A. Moreno, Timothy Ravasi, Jasmeen S. Merzaban, Pedro Ramos-Cabrer, Aitziber L. Cortajarena, Jürgen Kosel

**Affiliations:** 1 Division of Biological and Environmental Sciences and Engineering, King Abdullah University of Science and Technology, Thuwal, Jeddah, 23955-6900 Saudi Arabia; 2 Center for Cooperative Research in Biomaterials (CIC biomaGUNE), Basque Research and Technology Alliance (BRTA), Paseo de Miramon 182, 20014 Donostia San Sebastián, Spain; 3 Division of Computer, Electrical and Mathematical Sciences and Engineering, King Abdullah University of Science and Technology, Thuwal, Jeddah, 23955-6900 Saudi Arabia; 4 Ikerbasque, Basque Foundation for Science, Mª Díaz de Haro 3, 48013 Bilbao, Spain; 5 IMDEA Nanociencia and Nanobiotechnology Unit Associated to Centro Nacional de Biotecnología (CNB-CSIC), Campus Universitario de Cantoblanco, 28049 Madrid, Spain

**Keywords:** Cell tracking, Magnetic resonance imaging, Iron-iron oxide, Core–shell, Nanowires, Cell labeling, T_2_ contrast

## Abstract

**Background:**

Identifying the precise location of cells and their migration dynamics is of utmost importance for achieving the therapeutic potential of cells after implantation into a host. Magnetic resonance imaging is a suitable, non-invasive technique for cell monitoring when used in combination with contrast agents.

**Results:**

This work shows that nanowires with an iron core and an iron oxide shell are excellent materials for this application, due to their customizable magnetic properties and biocompatibility. The longitudinal and transverse magnetic relaxivities of the core–shell nanowires were evaluated at 1.5 T, revealing a high performance as T_2_ contrast agents. Different levels of oxidation and various surface coatings were tested at 7 T. Their effects on the T_2_ contrast were reflected in the tailored transverse relaxivities. Finally, the detection of nanowire-labeled breast cancer cells was demonstrated in T_2_-weighted images of cells implanted in both, in vitro in tissue-mimicking phantoms and in vivo in mouse brain. Labeling the cells with a nanowire concentration of 0.8 μg of Fe/mL allowed the detection of 25 cells/µL in vitro, diminishing the possibility of side effects. This performance enabled an efficient labelling for high-resolution cell detection after in vivo implantation (~ 10 nanowire-labeled cells) over a minimum of 40 days.

**Conclusions:**

Iron-iron oxide core–shell nanowires enabled the efficient and longitudinal cellular detection through magnetic resonance imaging acting as T_2_ contrast agents. Combined with the possibility of magnetic guidance as well as triggering of cellular responses, for instance by the recently discovered strong photothermal response, opens the door to new horizons in cell therapy and make iron-iron oxide core–shell nanowires a promising theranostic platform.

## Background

Over the last years, nanomaterials have been widely investigated for improving the diagnosis of diseases and their treatments [1]. Thanks to their structural, chemical, optical or mechanical properties, nanomaterials provide the ability to interact with cells in different ways and with remote control mechanisms [2–4]. Due to the importance of a very early-stage detection of disease, biomedical imaging has become an invaluable tool for both scientific and clinical applications [5]. In particular, magnetic resonance imaging (MRI) is a powerful tool, allowing the non-invasive and real-time monitoring of dynamic processes in living cells and organisms. Contrast agents (CAs) are often utilized in MRI, to better discern tissues of similar magnetic properties, by shortening the transversal (T_2_) and/or the longitudinal (T_1_) relaxation times of the nearby water protons at the region of interest [6, 7]. Commercially available T_1_ CAs are paramagnetic complexes, usually gadolinium (Gd^3+^) chelates [8], while T_2_ CAs are mostly based on iron oxide (Fe_x_O_y_) nanoparticles (NPs) [9, 10]. Fe_x_O_y_ NPs are superparamagnetic and exhibit a magnetization proportional to an external magnetic field, which generates local magnetic field inhomogeneities and accelerates the dephasing of the surrounding protons’ spins [7]. Limitations such as the passive accumulation of Fe_x_O_y_ NPs in the liver and spleen [11, 12] together with signal attenuation (low signal-to-noise ratio) [13, 14], strong blooming effect [15], and high background interference (low specificity) [16] have restricted their clinical application [17–19]. Nevertheless, T_2_ CAs are highly appreciated for research purposes in relevant applications such as cell tracking studies [20]. Besides, a new generation of particles is being developed to tackle the previously encountered issues [21, 22] and functionalities are dramatically expanded by imaging and guiding the particles, i.e. magnetic resonance navigation [23]. In order to be an efficient T_2_ CA, a nanomaterial should possess a large transversal magnetic relaxivity constant, r_2_, which is proportional to the effective magnetic moment of the material [24]. This requirement places nanowires (NWs) composed of iron (Fe) in a prime position, due to their high saturation and remanent magnetization values, the latter originating from the strong shape anisotropy [25, 26]. NWs can be grown in nanoporous templates by electrochemical deposition, which is a simple and efficient method that provides accurate control of the length and diameter [27–29].

Previously, the performance of Fe-based NWs and multisegmented Fe/gold NWs was assessed, where both types of NWs appeared to be promising T_2_ CAs [30]. Similarly, an aqueous suspension of coated nickel NWs exhibited good performance for T_2_ contrast with r_2_ values similar to those of commercial Fe_x_O_y_ NPs [31]. However, genotoxicity and cytotoxicity effects have been reported in nickel-containing particles [32].

In this paper, we investigate the performance of Fe–Fe_x_O_y_ core–shell NWs as tunable T_2_ CAs. We utilize them for MRI cell tracking, which is a rapidly advancing medical method since the earliest studies of stem cell detection [33–35]. In order to employ the therapeutic potential of cells, their precise location, and the dynamics of their migration and differentiation after implantation into the host must be monitored and understood. Fe_x_O_y_-based nanomaterials are among the most used systems for labeling of cells for in vivo tracking experiments [20, 34, 36]. One of the key aspects of cell tracking studies is the sensitivity to detect a small number of cells after implantation. For this task, the strong magnetization [37] and biocompatibility [38–40] of Fe-Fe_x_O_y_ core–shell NWs are foreseen as advantageous characteristics.

In this work, we studied the actuation of Fe-Fe_x_O_y_ core–shell NWs for T_2_ contrast at representative clinical (1.5 T) and preclinical (7 T) fields, and at a different level of oxidation, and surface coatings. The amount of NWs interacting with the cells was spectrometrically quantified and the labeling sensitivity was assessed by in vitro T_2_-weighted images of the cells embedded in an agar gel. In vivo MRI cell tracking studies were carried out at 11.7 T, implanting the labeled cells into mouse brains.

## Results and discussion

### Nanowire characterization

**Fig. 1:**
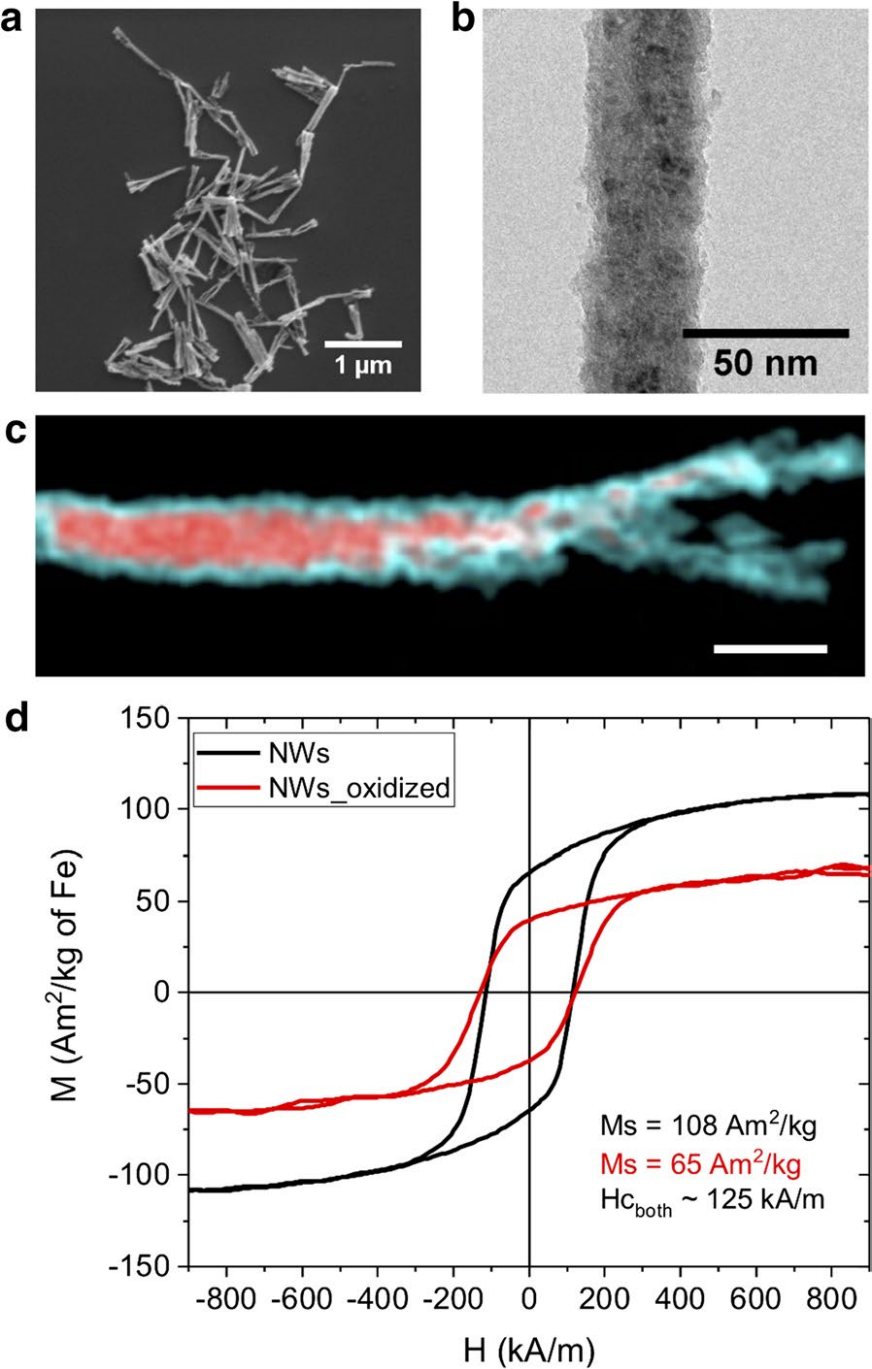
Characterization of Fe-based NWs. **a** SEM image of NWs. **b** TEM image of NWs. **c** EELS mapping of a single NW. STEM image with superimposed Fe (red) and oxygen (blue) color mix map of NWs with native oxidation layer. The scale bar corresponds to 50 nm. **d** Magnetization curves of Fe-based NWs with native oxide layer (black curve) and oxidized NWs (red curve) (Magnetization M is presented as Am^2^/kg of Fe, M_S_ is the saturation magnetization and H_C_ the coercive field, n = 3)

NWs composed by Fe were produced with an average diameter of 30 to 40 nm and an average length of 0.7 ± 0.16 μm (n = 100), as illustrated in Fig. 1a, b. Since NWs are exposed to air, water, sodium hydroxide, ethanol, etc., oxidation of their surface is unavoidable. The oxidation creates core–shell NWs with an oxide layer that is typically 4–10 nm thick [39] composed of mainly Fe_2_O_3_ and Fe_3_O_4_ [37, 40], and has a significant contribution to the biocompatibility [37], functionalizability, and magnetic properties of the NWs [39–43]. The electron energy loss spectroscopy (EELS) composition map in Fig. 1c confirms a core–shell structure with an Fe core (red color) surrounded by an FexOy shell (blue color).

In order to increase the thickness of the oxidation shell and thereby reduce the Fe core, a sample of NWs was subjected to oxidizing conditions (NWs_oxidized). The level of oxidation was evaluated by scanning transmission electron microscopy (STEM) and EELS in NWs that were placed in oxidizing conditions, and NWs that remained in ethanol all the time at room temperature. In both cases, the average relative concentrations of oxygen and Fe were measured in a specific region of different single NWs (Additional file 1: Figure S1) showing a mix between both elements in the oxidized shell and Fe in the core, (Additional file 1: Table S1, n = 3 for each condition). The oxygen/Fe ratio indicates the change in the thickness of the oxidation layer, which was then expressed as the percentage of oxygen and Fe in each NW analyzed. The average percentage of oxygen was found to be 33.7 ± 7% for the treated NWs and 17.7 ± 3% for the control NWs with a native oxide layer.

The magnetization curves of both samples of NWs with different oxidation level are shown in Fig. 1d. From these, the values of the saturation magnetization (M_S_) are 108 Am^2^/kg of Fe for the NWs with a native oxide layer and 65 Am^2^/kg of Fe for the oxidized sample. The M_S_ of the non-oxidized NWs is higher than the M_S_ reported for Fe_2_O_3_ and Fe_3_O_4_-based NPs [44–46], which increases the performance for T_2_ contrast [24], due to a more efficient induction of field inhomogeneity and a larger area of influence [21]. Yet, the measured M_S_ values of both NW samples are lower than previously reported ones for longer Fe-based NWs inside of the alumina template (143 Am^2^/kg of Fe) [47], and below the M_S_ of bulk Fe at room temperature (218 Am^2^/kg) [48].

The NWs_oxidized showed approximately 40% reduction in the M_S_ compared to NWs with native oxide layer, which is directly related to the increase in the thickness of the Fe_x_O_y_ shell in the oxidized sample [37]. It could also be attributed to corrosion (Fe ions lost in the hydrogen peroxide oxidation step) [49]. If assuming a linear relationship between the oxide shell volume and the M_S_ reduction, it is easy to show that this 40% reduction in M_S_ corresponds to an increase of shell thickness of around 4 nm in each NW. The coercive field was ~ 125 kA/m for both formulations since the NWs take random orientations, compared to NWs inside a template.

In this study, NWs with a native oxide layer and NWs_oxidized were coated with (3-aminopropyl) triethoxysilane, APTES [50], (APTES-NWs and APTES-NWs_oxidized, respectively) and NWs with native oxide layer were coated with bovine serum albumin, BSA, (BSA-NWs) as previously reported [51, 52]. The presence of both coating agents around the Fe-based NWs has been previously confirmed [38, 40, 52], and has been shown to improve the biocompatibility and colloidal properties of the NWs with a reduction of agglomeration and enhancement of dispersion [40, 43]. Such improvement can be attributed to electrostatic interactions supported by a zeta potential of − 58 mV [43] and − 17.4 mV [52] for the BSA-NWs and APTES-NWs, respectively. Furthermore, the presence of free chemical groups of both, BSA and APTES enables further functionalization of the NWs [40, 43, 51, 53].

### Relaxivity measurements

The longitudinal (r_1_) and transversal (r_2_) relaxivities, as well as r_2_/r_1_ ratios, for different formulations of NWs were calculated from the relaxation times measured on an MR relaxometer at 1.5 T (Additional file 1: Figure S2, Table S2) in a preliminary characterization. Linear fitting of the relaxation rates (R_1_ = 1/T_1_, Additional file 1: Figure S2A and R_2_ = 1/T_2_, Additional file 1: Figure S2B), as a function of Fe concentration, was performed using:

1 $${R}_{\mathrm{1,2}}\left({s}^{-1}\right)={R}_{\mathrm{10,20}}+{r}_{\mathrm{1,2}}\left[CA\right],$$

where R_10,20_ are the relaxation rates of the solvent, R_1,2_ the relaxation rates of NW solutions and [CA] the concentration of CA. Considering the elevated r_2_ values and r_2_/r_1_ ratios obtained for all Fe-based NW formulations, and their practically negligible effect on T_1_, these materials can be considered as competitive T_2_ CAs (Additional file 1: Table S2) [7]. Moreover, the performance of Fe-based NWs for T_2_ contrast (r_2_ ranging from 35 to 70 s^−1^ M^−1^) is close to commercial Fe_x_O_y_-based CAs such as Endorem™ (r_2_ ~ 41 s^−1^ mM^−1^) and Resovist (r_2_ ~ 61 s^−1^ mM^−1^) at 1.5 T [54].

Initial exploration of r_2_ relaxivities of selected formulations of Fe-based NWs at preclinically (7 T) relevant magnetic field (Table 1), showed that r_2_ values varied with time. Therefore, a set of experiments was performed measuring r_2_ for 160 min, with a lapse of 20 min between each time point. The first experimental r_2_ value was obtained at t = 20 min (time required for securing the samples inside the scanner, tuning the system, and acquire the required MR images). An exponential decay of r_2_ with time was observed, and the estimated r_2_ values (Table 1) at time zero (beginning of the magnetic field exposition) for each sample were obtained after fitting the r_2_ vs. time plots to a mono-exponential decay equation (Additional file 1: Figure S3A). A semilogarithmic representation of the r_2_ values over time (Additional file 1: Figure S3B), as well as the half time signal decay (t_1/2_ in Table 1) for each of the NW formulations, confirm the mono-exponential decay of the r_2_. The Fe-based NWs showed an r_2_ comparable to the one reported for the commercial formulation Feridex® at 7 T (166.71 s^−1^ mM^−1^)[55]. A reduction in r_2_ values is observed, when comparing non-oxidized and oxidized NWs (NWs vs. NWs_oxidized (non-coated formulations) and APTES-NWs vs. APTES-NWs_oxidized (coated formulations)). This decrease in r_2_ of ~ 40% and ~ 30% for the non-coated and coated formulations, respectively, is related to the 40% decrease in the M_S_, due to the oxidation of the NWs, which, in turn, reduces NW agglomeration. It has been reported that different factors, such as chemical composition, particle size and geometry, agglomeration, and polydispersity directly interfere with r_2_ [53, 56–61]. In fact, low levels of aggregation may lead to an increase of relaxivity, as aggregated particles in solution induce larger alterations of the local magnetic field (inhomogeneities). On the other hand, larger levels of aggregation may lead to particle sedimentation; hence, the relaxivity of the bulk solution will be reduced (approaching the one of the solvent) [62]. The NWs r_2_ was also affected by the addition of coating agents, where a slight decrease in the r_2_ was found when comparing the NWs coated with APTES with the non-coated NWs (NWs vs. APTES-NWs and NWs_oxidized vs. APTES-NWs_oxidized). This decrease is attributed to an increase in steric stability [64] and indicates that the coating of the NWs produces a reduction in agglomeration, which affects the r_2_ values [52, 53]. Besides, since the NWs behave as single magnetic domain structures, the magnetic inhomogeneities affecting the relaxation time of the surrounding protons are generated only at the tips of the NWs with no contribution of the NW body [63–65]. The BSA-NWs present the lowest r_2,_ with a value at least three times lower than the other formulations. This reduction in the r_2_ can be partially attributed to the large volume of BSA that not only prevents aggregation of particles by increasing the distance between them, by steric hindrance [51], but also increases the distance between the NWs and the surrounding protons, reducing the magnetic inhomogeneity [66, 67]. Besides, BSA-NWs presented the largest half time of the r_2_ signal, which is related to higher colloidal stability.

**Table 1:** Transversal (r_2_) relaxivity values and relaxivity half time decay of different nanowire formulations at 7 T

Formulation	r_2_ (s^−1^ mM^−1^)	t_1/2_ (min)
NWs	281	23.6
APTES-NWs	221	34.3
NWs_oxidized	176.8	31.7
APTES-NWs_oxidized	155	26.6
BSA-NWs	50.1	51.4

Although all samples were sonicated before the measurements, the magnetic field of the imaging system at 7 T enhanced the NW aggregation as exemplified in Additional file 1: Figure S4. There is an abrupt decrease (more than 70%) of the r_2_ value (from 55.5 to 17.1 s^−1^ mM^−1^) in the samples that remained inside the equipment throughout the measurement time (60 min, Additional file 1: Figure S4A) compared to the lower decrease of r_2_ (from 67.4 to 51.7 s^−1^ mM^−1^, ~ 23%) observed for the NWs that were placed in the MRI system only at the measurement time points (0 min and 60 min, Additional file 1: Figure S4B). The NW aggregation and/or sedimentation enhancement by the magnetic field of the MRI equipment occurs through the NW alignment and attractive forces [59, 61]. Several Fe_x_O_y_-based nanomaterials have been observed to cluster as a function of time in the presence of a magnetic field [68, 69].

**Fig. 2:**
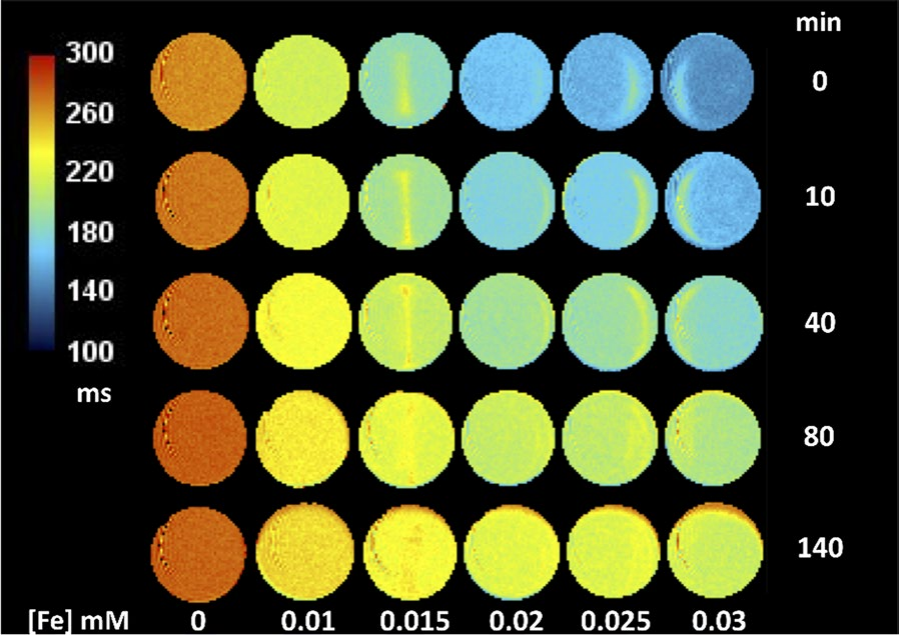
T_2_ parametric maps (2D slices showing the section) of tubes filled with APTES-NWs aqueous solutions at five different concentrations (x-axis), acquired at five selected time points (y-axis) in a 7 T MRI. The first column at the left side contains HPLC grade water and was used as a control

The T_2_ parametric map of APTES-NWs (Fig. 2) shows a NW concentration dependence of the R_2_. A sudden decrease in the r_2_ during the first 40 min is observed similar to the decay observed in Additional file 1: Figure S4. Additionally, after 160 min, the r_2_ of the most diluted NW solution tended towards the r_2_ value of HPLC grade water, used as control. As explained above, this decay of r_2_ values is attributed to aggregation and sedimentation of NWs in suspension, which is more pronounced in presence of high magnetic fields. Variations in the relaxation times, and therefore the colors, are visible in some of the tubes and can be explained by the inhomogeneous distribution of the NWs in suspension given by the aggregation and sedimentation.

### Magnetic resonance imaging of nanowires internalized in breast cancer cells

Studies on the cellular uptake and degradation of NWs with similar dimensions as well as the cellular internalization and biocompatibility of Fe-based NWs (identical to the ones used in this study) coated with APTES and BSA in MDA-MB-231 cancer cells have been previously reported [40, 43, 70, 71]. Cellular internalization of NWs is a continuous process that starts upon contact between NWs and cells [52], and takes place through the activation of the integrin-mediated phagocytosis pathway [26, 72]. Here, inductively coupled plasma mass spectrometry (ICP-MS) measurements were performed to quantify the NWs interacting with the cells. A total amount of ~ 35 pg of Fe/cell was found for cells incubated with APTES-NWs and ~ 117 pg Fe/cell for the cells incubated with BSA-NWs for 24 h without transfection agent. These values represent an internalization of ~ 40% and ~ 76% from the total amount of APTES-NWs and BSA-NWs added to the cells, respectively, corroborating our previous findings. It is worth mentioning that these values refer not only to internalized NWs, but also to NWs embedded in or adsorbed onto the extracellular structures surrounding the plasmatic membrane, and which can be present even after several washing steps.

**Fig. 3:**
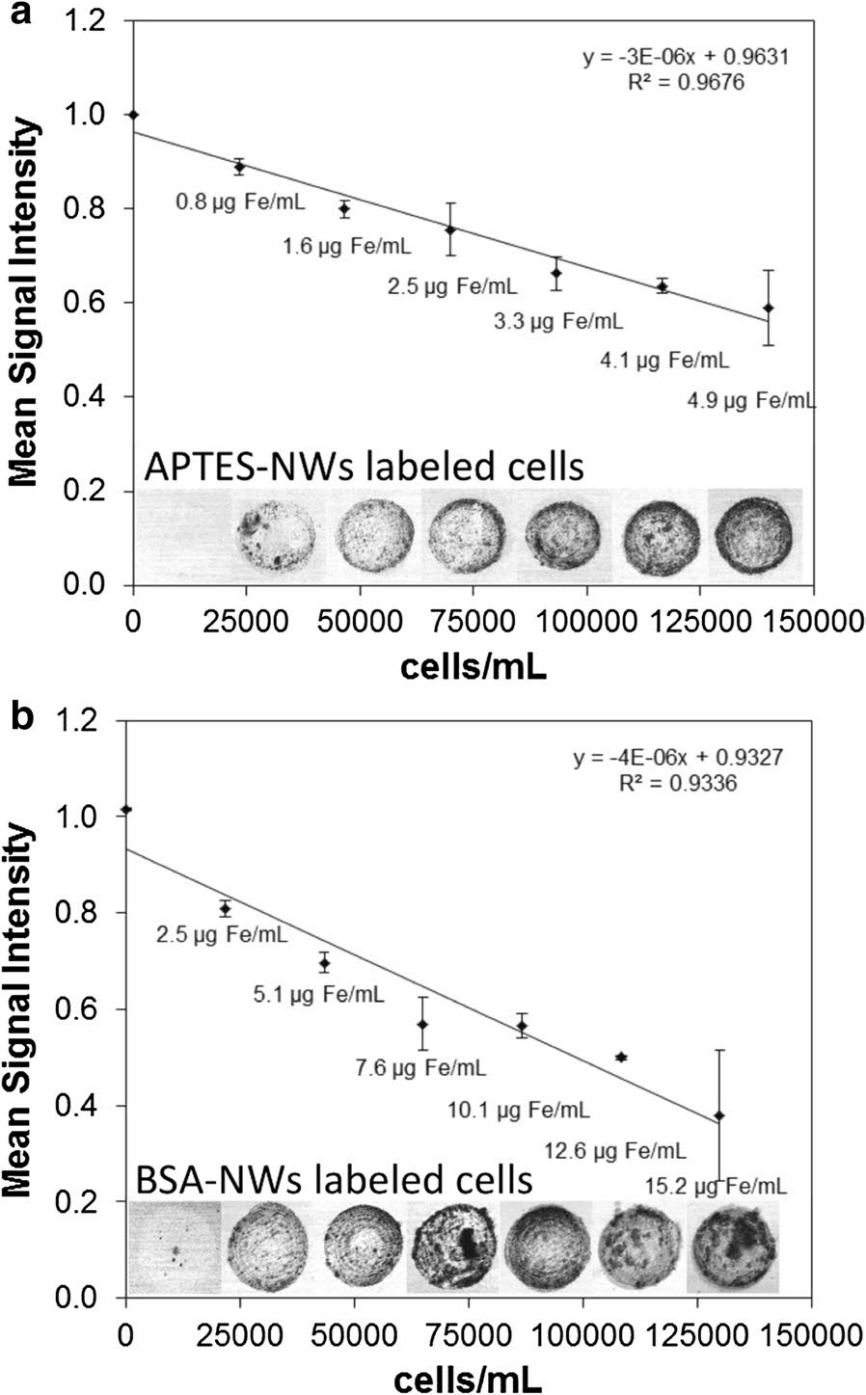
Variation of mean signal intensity (± SD, n = 3) with the concentration of MDA-MB-231 cells labeled with APTES-NWs (**a**) or BSA-NWs (**b**). Averaged T_2_* weighted images (acquired at 7 T) of cells are presented at the bottom of the plots, for each cell concentration

In vitro studies of Fe NWs internalized in breast cancer cells were performed to evaluate their efficiency as T_2_ cell labeling CAs (Fig. 3). Serial dilutions from a suspension of MDA-MB-231 cells labeled with APTES-NWs (12 μg of Fe/mL) or BSA-NWs (20 μg of Fe/mL) were sandwiched in an agar gel and a cell suspension of non-labeled cells was used as a negative control (n = 3). 3D-Gradient Echo T_2_* weighted MRI images were generated for each gel 24 h after implantation in a 7 T MRI equipment, from which a single 2D image was constructed by minimal intensity projection of all stacked slices that showed the presence of cells. The integrated T_2_* signals of each of these 2D projections were measured for each dilution of cells labeled with NWs and control (non-labeled) cells and the signal intensity was normalized to the value observed for agars. Averaged T_2_* weighted images of different concentrations of labelled cells in agar are presented together with the mean (± SD) signal intensity, for APTES-NWs (Fig. 3a) or BSA-NWs (Fig. 3b). In T_2_* weighted images, signal intensity (*S*) is exponentially dependent on the relaxation rate R_2_*:

2 $$S={S}_{0}exp(-TE {{R}_{2}}^{*})$$

where *S*_*0*_ is the value of S extrapolated to a null echo time TE = 0, i.e. in absence of T_2_* relaxation. Thus, according to Eqs. 1 and 2, *S* is directly proportional to the concentration of CA (Fe inside the cells), so the larger the number of labeled cells, the larger the *R*_2_* value (Eq. 1), and the lower the mean signal intensity (Eq. 2).

Overall, cell labeling with both formulations show a similar trend in the reduction of the mean signal intensity of MDA-MB-231 cells (Fig. 3). Although BSA-NWs showed an r_2_ ca. 5 times lower than the APTES-NWs, a similar number of cells with similar mean signal intensities were observed when comparing wells enclosing cells with the same Fe concentration for both formulations. This can be partially explained by the higher concentration of the BSA-NWs added to the cells and the higher cellular internalization of this formulation. A minimum cell concentration of approx. 25 cells/µL was detected using a NW concentration of 0.8 μg of Fe/mL and 2.5 μg of Fe/mL for the cells labeled with APTES-NWs (Fig. 3a) and BSA-NWs (Fig. 3b), respectively. T_2_* weighted images show a signal intensity distributed evenly throughout the whole well, indicating that labelled cells are distributed with minor aggregation. Hypointense regions at the edges of the wells given by cell accumulation were unavoidable, due to how the wells were produced in the gel. Micro air bubbles inside of the agar gel are seen as big black spots in T_2_* weighted images (Fig. 3a, b) and could influence the signal intensity measured in each gel (false positives). This fact is reflected in the SD at each cell concentration. The resolution of MRI cell detection depends on many factors such as the r_2_ value of the CA, the amount of internalized nanomaterial per cell, the MRI protocol, scanner specifications, and has reached the point of single-cell detection using Fe_x_O_y_-based nanoparticle labels [73]. The limit of cell detection with the core–shell Fe-Fe_x_O_y_ NWs was not elucidated in our experimental setup. Nevertheless, the results show the efficiency and sensitivity of Fe-based NWs as T_2_ CAs for cellular detection in systems simulating the tissue environment. The BSA-NWs were selected for further studies, due to their higher internalization compared to the APTES-NWs.

**Fig. 4:**
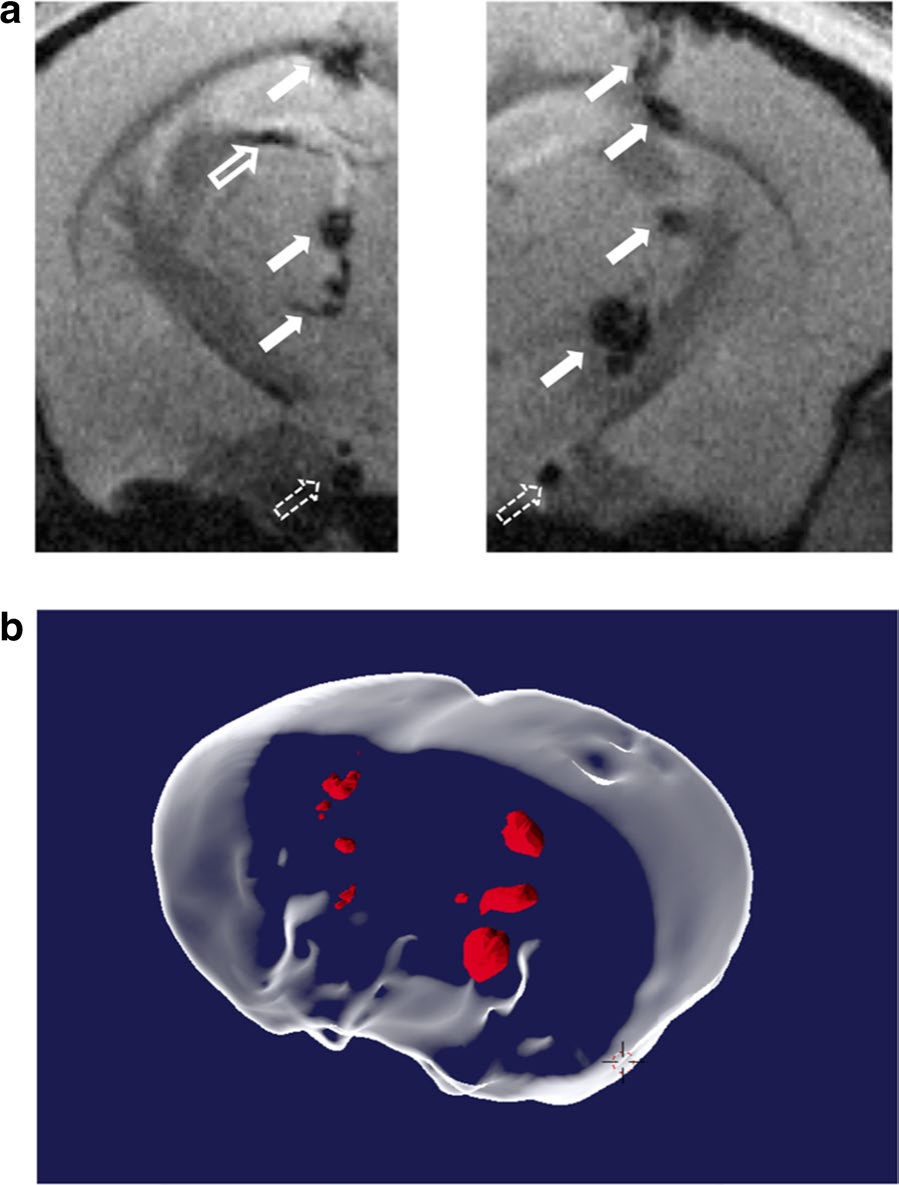
Detection of MDA-MB-231 cells labeled with BSA-NWs implanted in brains of living mice at 11.7 T. **a** Axial MR images of the brain of a mouse immediately after implantation of MDA-MB-231 cells labeled with BSA-NWs. Circa 10 cells were implanted on the left hemisphere (left image), while circa 100 cells were implanted in the right hemisphere (right image). NW labeled cells are distributed along the track opened by the needle used for their implantation (solid arrows), and partially infiltrated in the brain ventricle (hollow arrow). Large blood vessels (dashed arrows) appear as false positives for cell deposits (lack of specificity is a known limitation for in vivo imaging of T_2_ CAs). **b** Surface rendered reconstruction of the 3D set of T_2_* weighted images of the mice brain acquired after implantation of NW labeled cells. The signal produced by the labeled cells appears in red

Finally*,* the sensitivity of MRI detection of Fe-based NWs-labeled cells was tested in vivo by injection of the BSA-NWs labeled cells after implantation in mice brains (n = 3, Fig. 4). Circa 10 cells (Fig. 4a left, 3 µl of a 3500 cells/ml solution) and ca. 100 cells (Fig. 4a right, 3 µl of a 35,000 cells/ml solution) that were previously labeled with BSA-NWs were injected in the left and right hemisphere of a mouse brain, respectively, and immediately imaged by MRI at 11.7 T. Cell deposits are visible along the channel opened by the injection needle (backflow of cells along the needle track is difficult to avoid), and some of the cells ended up in the ventricles as indicated by the hollow arrows in Fig. 4a. The presence of large blood vessels in the field of view (dashed arrows in Fig. 4a) highlights one of the main problems of the in vivo use of negative CAs, which is the lack of specificity between exogenous labeled cells and endogenous negative contrast (in this case lack of signal in vessels due to blood flow). Nevertheless, the labeling provided by BSA-NWs was so effective that the sensitivity of detection could be pushed to the limit of enabling the location of small clusters of labeled cells, as observed in Fig. 4a. A surface rendered reconstruction of the 3D set of all T_2_* weighted images of the mice brain is shown in Fig. 4b**,** where the signal generated by the cells containing NWs appear in red color.

**Fig. 5:**
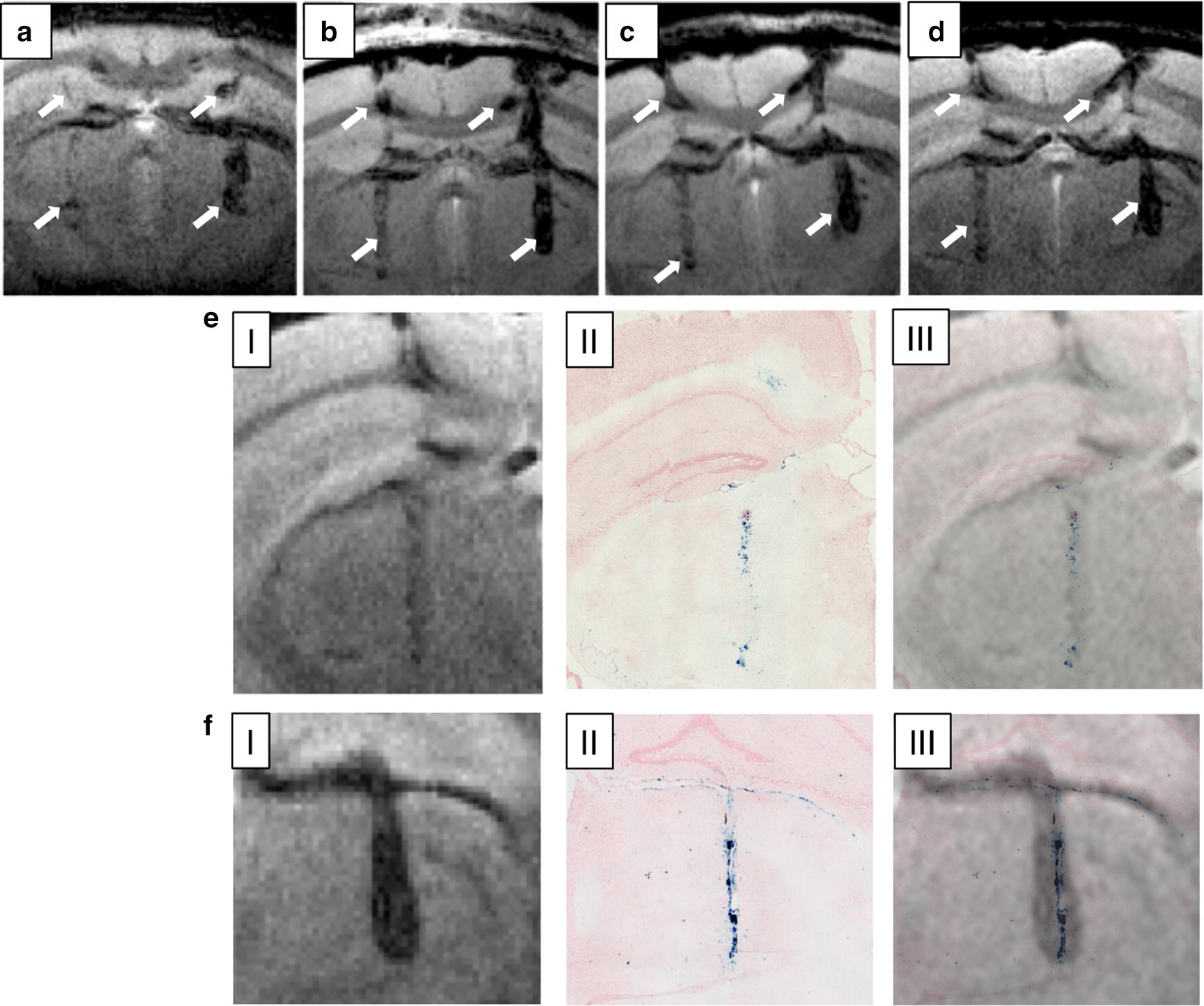
Distribution of MDA-MB-231 cells labeled with BSA-NWs after implantation in mouse brain at 11.7 T. Minimal intensity projections of all brain transverse 2D slices of the 3D image sets where BSA-NW labeled cells were present. Circa 10 cells were implanted on the left hemisphere (left side of the images), while circa 100 cells were implanted in the right hemisphere (right side of the images). Images were acquired immediately after implantation (**a**), and at days 10 (**b**), 20 (**c**), and 40 (**d**) post-implantation in living mouse. White arrows indicate the distribution of implanted cells along the injection needle tracks and into the corpus callosum. Correlation of mouse brain MRI images of implanted NW-labeled cells and optical images of histological slices of the same brain region stained with Perls’ Prussian blue for Fe detection 40 days post-implantation. Magnification of the left (**e**) and right (**f**) hemispheres showing MRI images (I), optical microscopy images of histological preparations (II), and overlay of both imaging methods (III). Perls’ Prussian blue stains NWs in blue due to the presence of Fe^3+^ in the oxide shell

MR imaging following the implantation of labeled cells in alive mice confirmed that the NWs produce an intense T_2_* contrast visible for several weeks (at least 6) after implantation (Additional file 1: Figure S5). High-resolution T_2_* weighted 3D images were acquired immediately after injection and 10, 20 and 40 days after implantation, showing the presence of NWs in a series of 2D image slice projections obtained from the 3D set. A detailed view of the cell location is presented in Fig. 5, where minimal intensity projections of all 2D slices with presence of NWs were constructed. Panels B-D in Fig. 5 shows that the labeled cells are distributed not only along the injection needle tracks but also traveled between injection sites along the corpus callosum. This could be potentially due to the activity of macrophages that engulfed injected cells, as part of the immune response to the implantation of exogenous cells in immune-competent mice rather than the active migration of the implanted cells. Our proof of principle in vivo studies do not provide information about the viability of the implanted cells and/or the integrity of the NWs throughout the weeks after the implantation. However, it has been reported that NWs with similar dimensions were observed inside of endosomes 24 h post-incubation with cells, and that a minimal fraction (~ 2%) of the NWs was dissolved intracellularly after 72 h due to the acidic environment of the lysosomal compartments in the cytoplasm [72]. Furthermore, large biodegradation of Fe nanoparticles was only observed after almost 1 month in a tissue environment and was partially determined by a decay on the M_S_ value [74]. A comparison between the NW-labeled cells detected by MRI (in vivo) and optical microscope images (ex vivo) of mice brain slices stained with Pearls’ Prussian blue for Fe detection, confirms the presence of Fe at the implantation site after 40 days and the correlation between NW labeling and the MRI T_2_*contrast (Fig. 5e, f). Throughout the 40 days and with 5 imaging sessions of 3 h exposure to the MRI’s magnetic field the labeled cells and NWs seem to be homogenously distributed, with no significant agglutination points.

The core–shell NWs have already been used for different therapeutic applications such as cancer cell killing through different modalities including pH-triggered drug delivery, magneto-mechanic actuation [40], and more recently photothermal therapy, where the NWs showed an outstanding heating efficiency [43]. Functionalization with targeting agents allowed the selective interaction with leukemic cells [38], and these NWs show potential for hyperthermia treatment and magnetic guiding [75, 76]. The possibility of using the NWs as MRI contrast agents opens the door for theranostics in diseases such as cancer, where different strategies can be combined for targeting (magnetic guiding and targeting agents) and for treatment, while following the process in a non-invasive manner. Specifically, MRI cell tracking with NWs can be applied for cell therapy in diverse pathologies including degenerative disorders, where pluripotent cells labeled with NWs can be magnetically guided and concentrated into the target site and their differentiation induced by photothermal, magnetic or mechanical responses of the NWs, as suggested in different publications [77–80].

## Conclusions

In this work, it is demonstrated the excellent performance of Fe-based NWs for T_2_ contrast in MRI detection of labeled cells. The capability of these Fe-Fe_x_O_y_ core–shell NWs with their high magnetization values to act as T_2_ (T_2_*) MRI CAs, as well as the effects of different variables on the T_2_ contrast such as level of oxidation of the NWs and surface coatings, were evaluated by measuring their r_2_ values at a preclinical relevant field. It was found that the r_2_ value of the NWs can be tailored by modifying the thickness of the oxide shell and by the addition of coating agents. T_2_* weighted images of MDA-MB-231 cells labeled with NWs embed in a tissue-like environment were produced testing two different NW formulations, whereby BSA coating not only enhanced dispersion and cellular internalization, but also showed a similar efficiency for MRI cell detection to the APTES coated NWs, despite having a smaller r_2_ value. Therefore, a lower concentration of BSA coated NWs can be employed to efficiently label the cells reducing, in turn, the possibility of toxic effects. In vitro, labeling of cancer cells with NWs at a concentration of 0.8 μg of Fe/mL enabled the detection of ~ 25cells/µL. In vivo studies showed that ca.10 NW-labeled cells could be located in the brain parenchyma of a mouse and were detectable for at least 40 days after implantation. The efficient and longitudinal cellular detection combined with the possibility of magnetic guidance of the NW labeled cells as well as triggering cellular responses opens the door to new horizons in cell therapy. These results together with the capacity of functionalization, magnetic responses, and the recently discovered qualities as a photothermal agent make the Fe-Fe_x_O_y_ core–shell NWs a promising theranostic platform.

## Methods

The main aspects of the methods are mentioned in this section. For detailed explanations of some of the procedures, see Additional file 1.

### Chemical reagents

Bovine serum albumin (BSA), 98% (3-aminopropyl) triethoxysilane (APTES), and 0.25% trypsin–EDTA solution were purchased from Sigma-Aldrich. 30% hydrogen peroxide was purchased from Electron Microscopy Sciences, Pennsylvania, USA. HPLC grade water (Sigma Aldrich) was used in all experiments if not indicated otherwise.

### Cell culture

The MDA-MB-231 cell line was purchased from American Type Culture Collections (Manassas, VA, USA). It was grown as a monolayer in Dulbecco’s Modified Eagle’s Medium (DMEM) supplemented with 10% fetal bovine serum (FBS), 2 mM L-glutamine, 100 units of penicillin/mL and 100 µg/mL of streptomycin. All reagents were purchased from GIBCO. Cells were maintained in an incubator at 37 °C in a humidified atmosphere of 95% air and 5% CO_2_.

### Preparation and characterization of nanowire formulations

Fe-Fe_x_O_y_ core–shell NWs were fabricated by chemical electrodeposition into nanoporous alumina membranes, as previously reported [27–29, 72]. The NWs’ length of ~ 700 nm was controlled by the deposition time (16 min). The NWs were released from the alumina template and collected with a magnetic rack (DynaMag™-2; Life Technologies, Carlsbad, CA, USA) and rinsed thoroughly 10 to 15 times with ethanol with sonication steps of 10 s in-between. Finally, the released NW´s were suspended in 1 mL of absolute ethanol. Thereafter, the sample of NWs was suspended in an aqueous solution of hydrogen peroxide 15% v/v and left in a thermomixer (Thermomixer comfort, Eppendorf) at 400 rpm and 40 °C for 3 weeks. The sample was then washed five times with absolute ethanol with sonication periods in between.

The morphology, length, and diameter of the NWs were investigated by SEM (Quanta 3D; FEI Company, Hillsboro, OR, USA) and TEM (Tecnai BioTWIN; FEI Company). Images were analyzed using the NIH software ImageJ and the NWs’ length was determined out of a hundred NWs from different samples. The chemical composition, as well as the level of oxidation of the NWs, were evaluated by STEM and EELS by comparing the treated samples against control NWs that remained all the time in absolute ethanol at room temperature (n = 3). To determine the oxidation level, the average relative concentrations of Fe and oxygen were measured in identical regions of different single NWs, and the resultant ratio was compared between the control and treated samples of NWs (n = 3). TEM studies in combination with EELS were performed using a Thermo Fisher (former FEI) Titan 80–300 TEM equipped with a Cs probe corrector and a Gatan Imaging Filter Quantum 966. The microscope was operated at 200 kV. The EELS maps were acquired in STEM mode as so-called spectrum imaging. The impact of increasing the oxide layer thickness on the magnetic properties of the NWs was evaluated by measuring the magnetic hysteresis loops with a LakeShore 7404 Vibrating Sample Magnetometer (n = 3) and the measurements were carried out on dried samples of dispersed NWs.

NWs were coated with APTES or BSA as described previously [40]. Briefly, NWs with native oxide layer and oxidized NWs were coated separately with APTES by adding 0.0946 g of APTES per each 100–300 mg of Fe to the NWs suspended in ethanol with a final volume of 5 mL and sonicated for 1 h. Since basic catalysis is required for the reaction, 200 μL of miliQ water and 10 μL of sodium hydroxide 1 M were added, followed by a second sonication period of 1 h. Finally, the NWs were washed five times with 1 mL absolute ethanol and stored at room conditions in an Eppendorf tube. For BSA coating, NWs with native oxide layer were suspended in 10 mM phosphate buffer pH 7.4 (PB), to a final volume of 2.5 mL in a glass vial and added with 0.8 mg of BSA/mg of Fe, followed by 1.5 of sonication. The BSA coated NWs were washed three times with phosphate buffer and stored at 4 °C in sterile conditions. All NW formulations were stored at room temperature and are stable for at least 12 months after the coating. The Fe concentration in each NW-formulation stock solution and in cell suspensions was quantified by triplicate using an ICP-MS (iCAP-Q instrument, Thermo Fisher Scientific).

### Transversal relaxivity and magnetic resonance imaging measurements

Transversal relaxivity measurements and in vitro imaging experiments were performed in a 7 T horizontal bore Bruker Biospec 70/30 USR scanner (Bruker Biospin, Ettlingen, Germany) using a BGA12-S imaging gradient set of 440 mT/m and a 40 mm inner diameter transmit/receive volumetric coil. A set of 5 solutions of each NWs formulation with Fe concentrations ranging from 0.01 to 0.03 mM were measured at 37 °C using a heated water blanket for temperature control. A tube with HPLC grade water was used as a control sample in all measurements. All samples were sonicated individually for 20 s before introducing them in the MRI equipment to ensure proper dispersion of the NWs. T_2_ relaxation time maps were acquired using a Multi-Slice Multi-Echo sequence with the following parameters: repetition time (RT) = 1500 ms, echo time (TE) = 20 ms, echo train of 24 equally spaced TE (20 to 480 ms, ∆TE = 20 ms), field of view (FOV) = 25.6 mm × 12.8 mm, image matrix = 256 × 128 points, one slice of 5 mm thickness and 2 averages. T_2_ relaxation times of each sample were measured at fixed intervals of ca. 20 min throughout 160 min. R_2_ relaxation rates were obtained as the reciprocal of T_2_ relaxation times (obtained by fitting MR signal decay with echo time to a mono-exponential function) and the corresponding r_2_ values were obtained using Eq. 1. T_2_ parametric maps were generated for each of the NW samples and were calculated by fitting pixel intensities of the multi-echo images to a mono-exponential decay on a pixel by pixel basis.

The capacity of NWs to perform as CA for cell tracking was assessed by labeling of breast cancer cells and studying them with MRI when placed in vitro and in vivo. Cell suspensions with a concentration of ~ 140,000 cells/mL were prepared from MDA-MB-231 cells labeled with APTES-NWs (12 μg of Fe/mL) and BSA-NWs (20 μg of Fe/mL). Five serial dilutions of the cells treated with each NW formulation were made (4200 to 700 cells /mL). Likewise, 3 dilutions of the non-treated cells were made with a concentration of 4200 to 1400 cells/mL). 30 μL of each dilution was applied to the wells of each one of the three agar gels previously prepared, which thereafter was left for 2 h at room temperature, enabling adsorption of the cells on the surface of the bottom of the wells. Subsequently, another agarose solution of 0.9% was prepared, left to reach just above physiological temperature and added to the three gels for filling the wells, therefore trapping the attached cells. Finally, gels were covered with Parafilm and left at 4 °C until imaging. For MRI acquisition, a Fast Low Angle Shot sequence was chosen to image 25 slices of 0.2 mm thickness. The FOV was 36 × 36 mm^2^, acquired with a matrix of 512 × 512 points, resulting in an in-plane resolution of 70 × 70 µm^2^. For imaging, the following parameters were used: TR = 500 ms, TE = 7.3 ms, and 20 averages. For the quantitative analysis, all the slices containing cells were used to calculate the minimal intensity projection taking into account that transfected cells produce a dark contrast on MR images, which were normalized by subtracting the agarose gel background signal. Finally, the integrated signal intensity was calculated for each sample from the minimal intensity projection image, and its dependence with the volume of labeled cells was obtained. Signal intensity was normalized to the value observed for agar to correct for factors such as distance and orientation of sample to the MR coil, magnetic field shimming, receiver gain of the system and other parameters that can vary from one experiment to the next.

In vivo imaging experiments were performed in a 11.7 T horizontal bore Bruker Biospec 117/16 USR scanner (Bruker Biospin, Ettlingen, Germany) using a BGA9-S imaging gradient set of 750 mT/m, and a 72 mm inner diameter transmit volumetric coil and actively decoupled mouse head surface coil for detection (both from Bruker Biospin). All animal experiments were approved by the local IACUC and local authorities and under the European Union Directive 2010/63/EU on the protection of animals used for scientific purposes. A total of 3 mice (c57bl/6, male, ~ 30 g) were used in the study. Briefly, after induction of anesthesia with isoflurane animals were positioned in a stereotaxic frame, the head was shaved, and an incision on the skin was performed along the brain midline exposing the skull of the animal. Two small holes were buried (one per hemisphere) in the bone using a dentistry drill. The needle of a Hamilton syringe of 10 µl containing the BSA-NWs-labeled cells solution, attached to the stereotaxic frame, was slowly introduced through each of the holes to a depth of 3.5 mm and raised 0.5 mm afterward (leaving a small reservoir to deposit the cells). A volume of 3 µl of BSA-NWs-labeled cell solutions with the concentration of 3500 cells/ml was injected in the left hemisphere, and 35,000 cells/ml was injected in the right hemisphere resulting in approximately 10 cells and 100 cells on the left and right side, respectively. The cells were deposited during 5 min in the brain of the animal leaving the needle in site for another five minutes after finishing the injection to minimized backflow of cells through the needle track. Finally, acrylic glue was used to close the bone holes, and animals were sutured and transferred to the MRI where images were acquired under isoflurane anesthesia. T_2_*-weighted MRI images were acquired using the following imaging parameters: 3D-Multi-Gradient-Echo sequence, TE = 4.35, 10.6, and 16.8 ms, TR = 80 ms, flip angle = 15, 6 averages, FOV = 12.8 × 9.6 × 6.4 mm (3 × FOV saturation bands), image matrix = 256 × 192 × 32 points (giving a spatial resolution of 50 × 50 × 200 µm) and effective bandwidth of 50 kHz. Images were acquired directly after implantation of cells and repeated after 10, 20, and 40 days.

### Histology

After the last MRI session, animals were euthanized and perfused with 20 ml of saline solution followed by 20 ml of paraformaldehyde 4%. Mice brains were extracted, rinsed with saline solution, and left in 4% paraformaldehyde overnight at 4 °C. The brains were then placed into 50 ml falcon tubes containing sucrose solution 20% and kept at 4 °C until they sunk to the bottom of the falcon. Finally, the brains were deeply frozen in methylbutane, cooled by dried ice, and cut in slices of 8 µm using a cryostat Leica CM3050S. Tissues were placed in glass slides and dried in a stove for 1 h. For Perls’ Prussian blue staining, slides were first immersed in distilled water for 30 s, then in a 6% Perls’ solution A (Potassium ferrocyanide trihydrate) plus Perls’ solution B (HCl) for 1 h. Slides were immersed in Fast Red for 2 min, washed with water and dehydrated with an ascending gradient of ethanol–water mixtures (ethanol 50%, 75%, 96%, 3–4 min in each mixture). Finally, slides were immersed into xylene for 3 min and covered with a DPX mount for histology.

## Supplementary information

**Additional file 1.** Supplementary figures describe the schematic of Fe-based NWs oxidation and its evaluation, the variation of the relaxation rates as a function of the Fe-based NW concentration at 1.5 T for different NW formulations, the decay of the r_2_ values obtained at 7 T across the time for different NW formulations, the influence of the NWs’ magnetization in the r_2_ decay across time, and four axial consecutive slices of 200 µm of thickness across the brain of a mouse implanted with BSA-NWs labeled cells. Supplementary tables describe the determination of the oxidation level of Fe NWs through elemental quantification, and the longitudinal (r_1_), transversal (r_2_) relaxivities and r_2_/r_1_ ratio of different nanowire formulations at 1.5 T. Supplementary methods describe the synthesis of nanowires, the magnetic characterization of nanowires, the quantification of iron nanowires, the relaxivity measurements at 1.5 T, the magnetization effect of iron nanowires at the 7 T, the preparation of cell suspensions for in vitro MRI detection and the preparation of agar gels for MRI imaging phantom studies.

## Data Availability

All data generated or analyzed during this study are included in this published article and its supplementary information files.

## Abbreviations

NW(s): Nanowire(s)
BSA: Bovine serum albumin
APTES: (3-aminopropyl)triethoxysilane
CA: Contrast agent
PBS: Phosphate buffer saline
Fe: Iron
Fe_x_O_y_: Iron oxide
FOV: Field of view
ICP-MS: Inductively coupled plasma mass spectrometry
MRI: Magnetic resonance imaging
T_1_: Longitudinal relaxation time
T_2_: Transversal relaxation time
TE: Time to echo
TEM: Transmission electron microscopy
TR: Time to repetition
SEM: Scanning electron microscopy
EELS: Electron energy loss spectroscopy
STEM: Scanning transmission electron microscopy
M_S_: Saturation magnetization
H_C_: Coercivity
r_1_: Longitudinal relaxivity
r_2_: Transversal relaxivity
R_1_: Longitudinal relaxation rate
R_2_: Transversal relaxation rate

## References

[1] Hilger I, Kaiser WA (2012). Iron oxide-based nanostructures for MRI and magnetic hyperthermia. Nanomedicine.

[2] Krishnan KM (2010). Biomedical nanomagnetics: a spin through possibilities in imaging, diagnostics, and therapy. IEEE Trans Magn.

[3] Golovin YI, Gribanovsky SL, Golovin DY, Klyachko NL, Majouga AG, Master AM (2015). Towards nanomedicines of the future: Remote magneto-mechanical actuation of nanomedicines by alternating magnetic fields. J Control Rel.

[4] Espinosa A, Di Corato R, Kolosnjaj-Tabi J, Flaud P, Pellegrino T, Wilhelm C (2016). Duality of iron oxide nanoparticles in cancer therapy: amplification of heating efficiency by magnetic hyperthermia and photothermal bimodal treatment. ACS Nano.

[5] Jacobs MA, Ibrahim TS, Ouwerkerk R (2007). MR Imaging: brief overview and emerging applications. RadioGraphics.

[6] Villaraza AJ, Bumb A, Brechbiel MW (2010). Macromolecules, dendrimers, and nanomaterials in magnetic resonance imaging: the interplay between size, function, and pharmacokinetics. Chem Rev..

[7] Na HB, Song IC, Hyeon T (2009). Inorganic nanoparticles for mri contrast agents. Adv Mater.

[8] Caravan P, Ellison JJ, McMurry TJ, Lauffer RB (1999). Gadolinium(III) chelates as MRI contrast agents: structure, dynamics, and applications. Chem Rev.

[9] Dias MHM, Lauterbur PC (1986). Ferromagnetic particles as contrast agents for magnetic resonance imaging of liver and spleen. Magn Reson Med.

[10] Semelka RC, Helmberger TKG (2001). Contrast agents for MR imaging of the liver. Radiology.

[11] Hamm B, Staks T, Taupitz M, Maibauer R, Speidel A, Huppertz A (1994). Contrast-enhanced MR imaging of liver and spleen: first experience in humans with a new superparamagnetic iron oxide. J Magn Reson Imag.

[12] Ferrucci JT, Stark DD (1990). Iron oxide-enhanced MR imaging of the liver and spleen: review of the first 5 years. Am J Roentgenol.

[13] Brisset J-C, Sigovan M, Chauveau F, Riou A, Devillard E, Desestret V (2011). Quantification of iron-labeled cells with positive contrast in mouse brains. Mol Imag Biol.

[14] Okuhata Y (1999). Delivery of diagnostic agents for magnetic resonance imaging. Adv Drug Deliv Rev.

[15] Hendrick RE, Mark HE (1993). Basic physics of MR contrast agents and maximization of image contrast. J Magn Reson Imag.

[16] Lee N, Hyeon T (2012). Designed synthesis of uniformly sized iron oxide nanoparticles for efficient magnetic resonance imaging contrast agents. Chem Soc Rev.

[17] Wang Y-XJ (2015). Current status of superparamagnetic iron oxide contrast agents for liver magnetic resonance imaging. World J Gastroenterol..

[18] Reimer P, Balzer T (2003). Ferucarbotran (Resovist): a new clinically approved RES-specific contrast agent for contrast-enhanced MRI of the liver: properties, clinical development, and applications. Eur Radiol.

[19] McCullough BJ, Kolokythas O, Maki JH, Green DE (2013). Ferumoxytol in clinical practice: Implications for MRI. J Magn Reson Imag.

[20] Hoehn M, Wiedermann D, Justicia C, Ramos-Cabrer P, Kruttwig K, Farr T (2007). Cell tracking using magnetic resonance imaging. J Physiol.

[21] Zhao Z, Zhou Z, Bao J, Wang Z, Hu J, Chi X (2013). Octapod iron oxide nanoparticles as high-performance T2 contrast agents for magnetic resonance imaging. Nat Commun.

[22] Lassenberger A, Scheberl A, Stadlbauer A, Stiglbauer A, Helbich T, Reimhult E (2017). Individually stabilized, superparamagnetic nanoparticles with controlled shell and size leading to exceptional stealth properties and high relaxivities. ACS Appl Mater Inter.

[23] Felfoul O, Becker AT, Fagogenis G, Dupont PE (2016). Simultaneous steering and imaging of magnetic particles using MRI toward delivery of therapeutics. Sci Rep.

[24] Koenig SH, Kellar KE (1995). Theory of 1/T1 and 1/T2 NMRD profiles of solutions of magnetic nanoparticles. Magn Reson Med.

[25] Hultgren A, Tanase M, Chen CS, Meyer GJ, Reich DH (2003). Cell manipulation using magnetic nanowires. J Appl Phys.

[26] Hultgren A, Tanase M, Felton EJ, Bhadriraju K, Salem AK, Chen CS (2005). Optimization of yield in magnetic cell separations using nickel nanowires of different lengths. Biotechnol Prog.

[27] Masuda H, Fukuda K (1995). Ordered metal nanohole arrays made by a 2-step replication of honeycomb structures of anodic alumina. Science.

[28] Nielsch K, Muller F, Li AP, Gosele U (2000). Uniform nickel deposition into ordered alumina pores by pulsed electrodeposition. Adv Mater.

[29] Pirota KR, Navas D, Hernandez-Velez M, Nielsch K, Vazquez M (2004). Novel magnetic materials prepared by electrodeposition techniques: arrays of nanowires and multi-layered microwires. J Alloys Compd.

[30] Shore D, Pailloux SL, Zhang J, Gage T, Flannigan DJ, Garwood M (2016). Electrodeposited Fe and Fe–Au nanowires as MRI contrast agents. Chem Commun.

[31] Banobre-Lopez M, Bran C, Rodriguez-Abreu C, Gallo J, Vazquez M, Rivas J (2017). A colloidally stable water dispersion of Ni nanowires as an efficient T2-MRI contrast agent. J Mater Chem B.

[32] Kasprzak KS, Sunderman FW, Salnikow K (2003). Nickel carcinogenesis. Mutat Res.

[33] Bulte JWM, Douglas T, Witwer B, Zhang S-C, Strable E, Lewis BK (2001). Magnetodendrimers allow endosomal magnetic labeling and in vivo tracking of stem cells. Nat Biotechnol.

[34] Bulte JWM, Duncan ID, Frank JA (2002). In vivo magnetic resonance tracking of magnetically labeled cells after transplantation. J Cereb Blood Flow Metab.

[35] Hoehn M, Küstermann E, Blunk J, Wiedermann D, Trapp T, Wecker S (2002). Monitoring of implanted stem cell migration in vivo: a highly resolved in vivo magnetic resonance imaging investigation of experimental stroke in rat. Proc Natl Acad Sci.

[36] Bulte JWM, Kraitchman DL (2004). Iron oxide MR contrast agents for molecular and cellular imaging. NMR Biomed.

[37] Ivanov YP, Alfadhel A, Alnassar M, Perez JE, Vazquez M, Chuvilin A (2016). Tunable magnetic nanowires for biomedical and harsh environment applications. Sci Rep.

[38] Alsharif NA, Martinez-Banderas A, Merzaban J, Ravasi T, Kosel J (2019). Biofunctionalizing magnetic nanowires toward targeting and killing leukemia cancer cells. IEEE Trans Magn.

[39] Meng-Meng S, Wen-Jing S, Hong B, Jun W, Wei-Lin W, Jun S (2010). Cytotoxicity and cellular uptake of iron nanowires. Biomaterials.

[40] Martínez-Banderas AI, Aires A, Teran FJ, Perez JE, Cadenas JF, Alsharif N (2016). Functionalized magnetic nanowires for chemical and magneto-mechanical induction of cancer cell death. Sci Rep.

[41] Reich DH, Tanase M, Hultgren A, Bauer LA, Chen CS, Meyer GJ (2003). Biological applications of multifunctional magnetic nanowires (invited). J Appl Phys.

[42] Alfadhel A, Kosel J (2015). Magnetic nanocomposite cilia tactile sensor. Adv Mater.

[43] Martinez-Banderas AI, Aires A, Quintanilla M, Holguin-Lerma JA, Lozano-Pedraza C, Teran FJ (2019). Iron-based core–shell nanowires for combinatorial drug delivery and photothermal and magnetic therapy. ACS Appl Mater Interfaces.

[44] Wang J, Sun J, Sun Q, Chen Q (2003). One-step hydrothermal process to prepare highly crystalline Fe_3_O_4_ nanoparticles with improved magnetic properties. Mater Res Bull.

[45] Zhao D-L, Zeng X-W, Xia Q-S, Tang J-T (2009). Preparation and coercivity and saturation magnetization dependence of inductive heating property of Fe_3_O_4_ nanoparticles in an alternating current magnetic field for localized hyperthermia. J Alloy Compd.

[46] Darezereshki E, Bakhtiari F, Alizadeh M, Behrad A, Ranjbar M (2012). Direct thermal decomposition synthesis and characterization of hematite (α-Fe_2_O_3_) nanoparticles. Mater Sci Semiconduct Process..

[47] Jose EP, Timothy R, Jürgen K (2017). Mesenchymal stem cells cultured on magnetic nanowire substrates. Nanotechnology.

[48] Tojkander S, Gateva G, Lappalainen P (2012). Actin stress fibers – assembly, dynamics and biological roles. J Cell Sci.

[49] Raphael MP, Christodoulides JA, Qadri SN, Simpkins BS, Byers JM (2010). Magnetic moment degradation of nanowires in biological media: real-time monitoring with SQUID magnetometry. Nanotechnology.

[50] Liu Y, Li Y, Li X-M, He T (2013). Kinetics of (3-Aminopropyl)triethoxylsilane (APTES) silanization of superparamagnetic iron oxide nanoparticles. Langmuir.

[51] Aires A, Ocampo SM, Cabrera D, de la Cueva L, Salas G, Teran FJ (2015). BSA-coated magnetic nanoparticles for improved therapeutic properties. J Mater Chem B.

[52] Margineanu MB, Julfakyan K, Sommer C, Perez JE, Contreras MF, Khashab N (2016). Semi-automated quantification of living cells with internalized nanostructures. J Nanobiotechnol.

[53] Arppe R, Nareoja T, Nylund S, Mattsson L, Koho S, Rosenholm JM (2014). Photon upconversion sensitized nanoprobes for sensing and imaging of pH. Nanoscale.

[54] Rohrer M, Bauer H, Mintorovitch J, Requardt M, Weinmann H-J (2005). Comparison of magnetic properties of MRI contrast media solutions at different magnetic field strengths. Invest Radiol.

[55] Kim H, Dae H-M, Park C, Kim EO, Kim D, Kim I-H (2011). A highly sensitive magnetite nanoparticle as a simple and rapid stem cell labelling agent for MRI tracking. J Mater Chem.

[56] Pöselt E, Kloust H, Tromsdorf U, Janschel M, Hahn C, Maßlo C (2012). Relaxivity optimization of a PEGylated iron-oxide-based negative magnetic resonance contrast agent for T2-weighted spin-echo imaging. ACS Nano.

[57] Ai H, Flask C, Weinberg B, Shuai X-T, Pagel MD, Farrell D (2005). Magnetite-loaded polymeric micelles as ultrasensitive magnetic-resonance probes. Adv Mater.

[58] Taktak S, Sosnovik D, Cima MJ, Weissleder R, Josephson L (2007). Multiparameter magnetic relaxation switch assays. Anal Chem.

[59] Vuong QL, Gillis P, Gossuin Y (2011). Monte Carlo simulation and theory of proton NMR transverse relaxation induced by aggregation of magnetic particles used as MRI contrast agents. J Magn Reson.

[60] Roch A, Gossuin Y, Muller RN, Gillis P (2005). Superparamagnetic colloid suspensions: Water magnetic relaxation and clustering. J Magn Magn Mater.

[61] Hak S, Goa PE, Stenmark S, Bjerkholt FF, Haraldseth O (2015). Transverse relaxivity of iron oxide nanocrystals clustered in nanoemulsions: Experiment and theory. Magn Reson Med.

[62] Peng E, Wang F, Xue JM (2015). Nanostructured magnetic nanocomposites as MRI contrast agents. J Mater Chem B.

[63] Vilanova Vidal E, Ivanov YP, Mohammed H, Kosel J (2015). A detailed study of magnetization reversal in individual Ni nanowires. Appl Phys Lett.

[64] Hertel R, Kirschner J (2004). Magnetization reversal dynamics in nickel nanowires. Phys B.

[65] Mohammed H, Moreno JA, Kosel J (2017). Advanced fabrication and characterization of magnetic nanowires. Magnet Magnetic Mater..

[66] Joshi HM, De M, Richter F, He JQ, Prasad PV, Dravid VP (2013). Effect of silica shell thickness of Fe_3_O_4_-SiOx core-shell nanostructures on MRI contrast. J Nanoparticle Res..

[67] LaConte LEW, Nitin N, Zurkiya O, Caruntu D, O'Connor CJ, Hu X (2007). Coating thickness of magnetic iron oxide nanoparticles affects R2 relaxivity. J Magn Reson Imaging.

[68] Chen DX, Sun N, Huang ZJ, Cheng CM, Xu H, Gu HC (2010). Experimental study on T2 relaxation time of protons in water suspensions of iron-oxide nanoparticles: Effects of polymer coating thickness and over-low 1/T2. J Magn Magn Mater.

[69] Muller RN, Gillis P, Moiny F, Roch A (1991). Transverse relaxivity of particulate MRI contrast media: from theories to experiments. Magn Reson Med.

[70] Perez JE, Contreras MF, Vilanova E, Ravasi T, Kosel J. Cytotoxicity and Effects on Cell Viability of Nickel Nanowires. In: International conference on biological, medical and chemical engineering (Bmce 2013). 2013. p. 178–84.

[71] Malak S, Yan MH, Guedeau-Boudeville MA, Conjeaud H, Garnier-Thibaud V, Boggetto N (2012). Interactions between magnetic nanowires and living cells: Uptake, toxicity and degradation. Abstr Papers Am Chem Soc.

[72] Perez JE, Contreras MF, Vilanova E, Felix LP, Margineanu MB, Luongo G (2015). Cytotoxicity and intracellular dissolution of nickel nanowires. Nanotoxicology..

[73] de Schellenberger AA, Kratz H, Farr TD, Lowa N, Hauptmann R, Wagner S (2016). Labeling of mesenchymal stem cells for MRI with single-cell sensitivity. Int J Nanomed.

[74] Mazuel F, Espinosa A, Luciani N, Reffay M, Le Borgne R, Motte L (2016). Massive intracellular biodegradation of iron oxide nanoparticles evidenced magnetically at single-endosome and tissue levels. ACS Nano.

[75] Shore D, Ghemes A, Dragos-Pinzaru O, Gao Z, Shao Q, Sharma A (2019). Nanowarming using Au-tipped Co(35)Fe(65) ferromagnetic nanowires. Nanoscale.

[76] Sharma A, Orlowski GM, Zhu YC, Shore D, Kim SY, DiVito MD (2015). Inducing cells to disperse nickel nanowires via integrin-mediated responses. Nanotechnology.

[77] Seo HI, Cho AN, Jang J, Kim DW, Cho SW, Chung BG (2015). Thermo-responsive polymeric nanoparticles for enhancing neuronal differentiation of human induced pluripotent stem cells. Nanomedicine..

[78] Dai R, Hang Y, Liu Q, Zhang S, Wang L, Pan Y (2019). Improved neural differentiation of stem cells mediated by magnetic nanoparticle-based biophysical stimulation. J Mater Chem B.

[79] Vissers C, Ming GL, Song H (2019). Nanoparticle technology and stem cell therapy team up against neurodegenerative disorders. Adv Drug Deliv Rev..

[80] Yi C, Liu D, Fong C-C, Zhang J, Yang M (2010). Gold nanoparticles promote osteogenic differentiation of mesenchymal stem cells through p38 MAPK pathway. ACS Nano.

