# Supplementary material for: Magnetic core–shell nanowires as MRI contrast agents for cell tracking
Source: J Nanobiotechnology. 2020 Mar 12;18:42. doi: 10.1186/s12951-020-00597-3 (PMC7069006; doi:10.1186/s12951-020-00597-3)
Supplement: Supplementary file 1 — Additional file 1. Supplementary figures describe the schematic of Fe-based NWs oxidation and its evaluation, the variation of the relaxation rates as a function of the Fe-based NW concentration at 1.5 T for different NW formulations, the decay of the r2 values obtained at 7 T across the time for different NW formulations, the influence of the NWs’ magnetization in the r2 decay across time, and four axial consecutive slices of 200 µm of thickness across the brain of a mouse implanted with BSA-NWs labeled cells. Supplementary tables describe the determination of the oxidation level of Fe NWs through elemental quantification, and the longitudinal (r1), transversal (r2) relaxivities and r2/r1 ratio of different nanowire formulations at 1.5 T. Supplementary methods describe the synthesis of nanowires, the magnetic characterization of nanowires, the quantification of iron nanowires, the relaxivity measurements at 1.5 T, the magnetization effect of iron nanowires at the 7 T, the preparation of cell suspensions for in vitro MRI detection and the preparation of agar gels for MRI imaging phantom studies. [file 12951_2020_597_MOESM1_ESM.docx]

Additional Information

Magnetic Core-Shell Nanowires as MRI Contrast Agents for Cell Tracking

Aldo Isaac Martínez-Banderas^†,1^, Antonio Aires^†, 2^, Sandra Plaza-García^2^, Lorena Colás^2^, Julián A. Moreno^3^, Timothy Ravasi^1^, Jasmeen S. Merzaban^1^, Pedro Ramos-Cabrer^2,4^*, Aitziber L. Cortajarena^2,4,5^*, Jürgen Kosel^3^*.

^1^Division of Biological and Environmental Sciences and Engineering, King Abdullah University of Science and Technology, Thuwal Jeddah, 23955-6900, Saudi Arabia.

^2^Center for Cooperative Research in Biomaterials (CIC biomaGUNE), Basque Research and Technology Alliance (BRTA), Paseo de Miramon 182, 20014, Donostia San Sebastián, Spain.

^3^Division of Computer, Electrical and Mathematical Sciences and Engineering, King Abdullah University of Science and Technology, Thuwal Jeddah, 23955-6900, Saudi Arabia.

^4^Ikerbasque, Basque Foundation for Science, Mª Díaz de Haro 3, Bilbao 48013, Spain.

^5^IMDEA Nanociencia and Nanobiotechnology Unit associated to Centro Nacional de Biotecnología (CNB-CSIC), Campus Universitario de Cantoblanco, Madrid, 28049, Spain.

^†^ These authors contributed equally to this work.

*Corresponding Authors. Email: pramos@cicbiomagune.es (P.R.C.), alcortajarena@cicbiomagune.es (A.L.C.), jurgen.kosel@kaust.edu.sa (J.K.)

**Additional Figures**

**
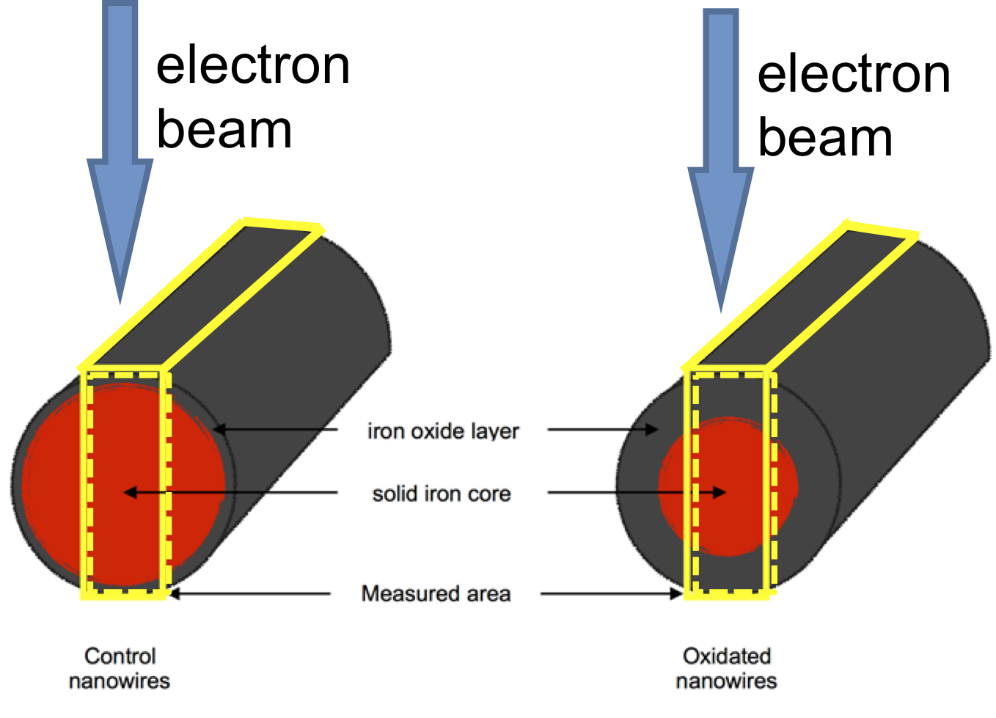
**

**Figure S1**. Schematic of Fe-based NWs oxidation and its evaluation. The level of oxidation of Fe NWs exposed to different oxidation conditions was determined by measuring the average relative concentrations of Fe and oxygen by using STEM and EELS.


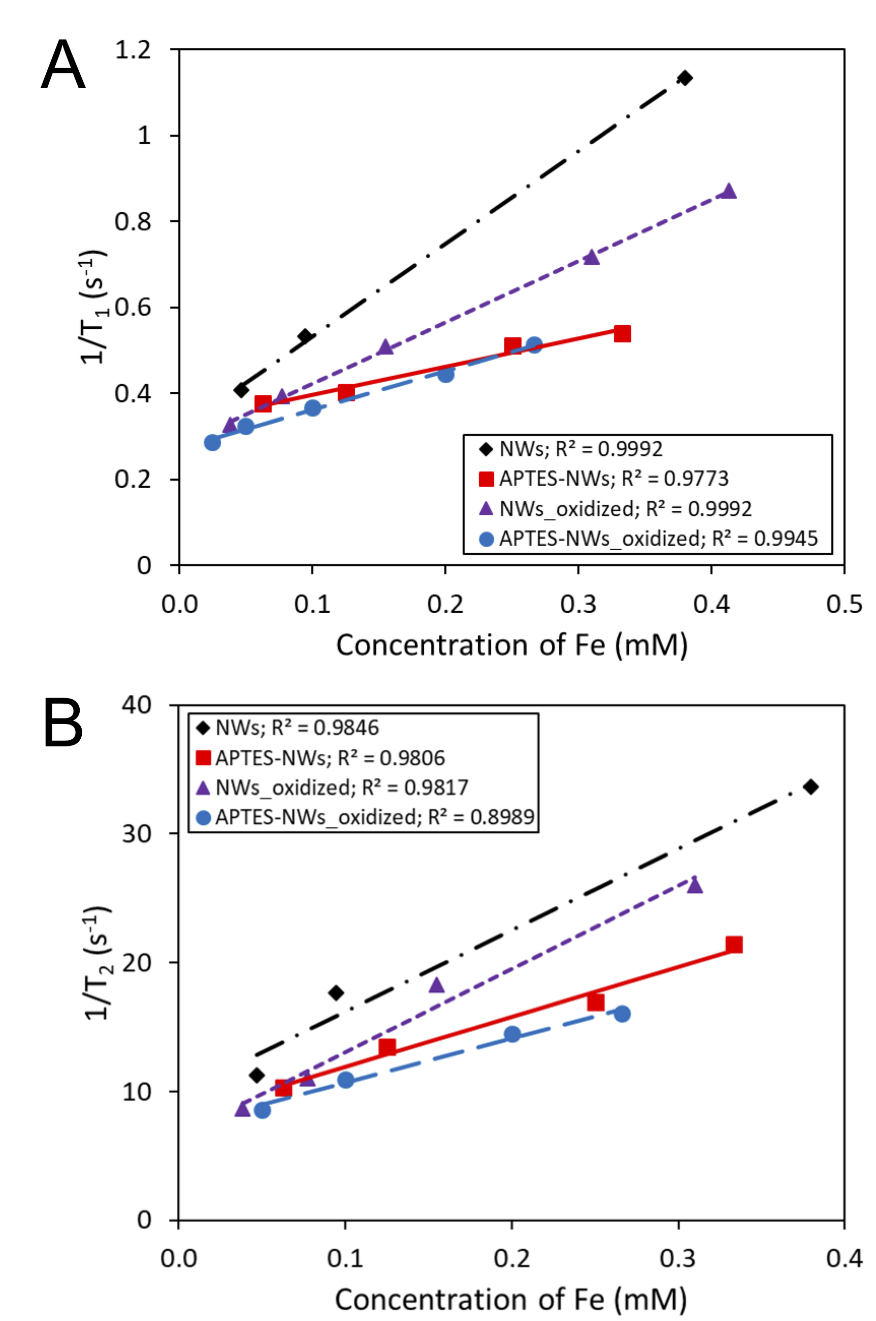


**Figure S2**. Variation of the relaxation rates as a function of the Fe-based NW concentration at 1.5 T for different NW formulations. (A) Variation of the longitudinal relaxation rate (R_1_= 1/T_1_) as a function of the NW concentration. The relaxivity r_1_ of each NW formulation was determined from the slope of each curve. (B) Variation of the transversal relaxation rate (R_2_= 1/T_2_) as a function of the NW concentration. The relaxivity r_2_ of each NW formulation was determined from the slope of each curve.

**
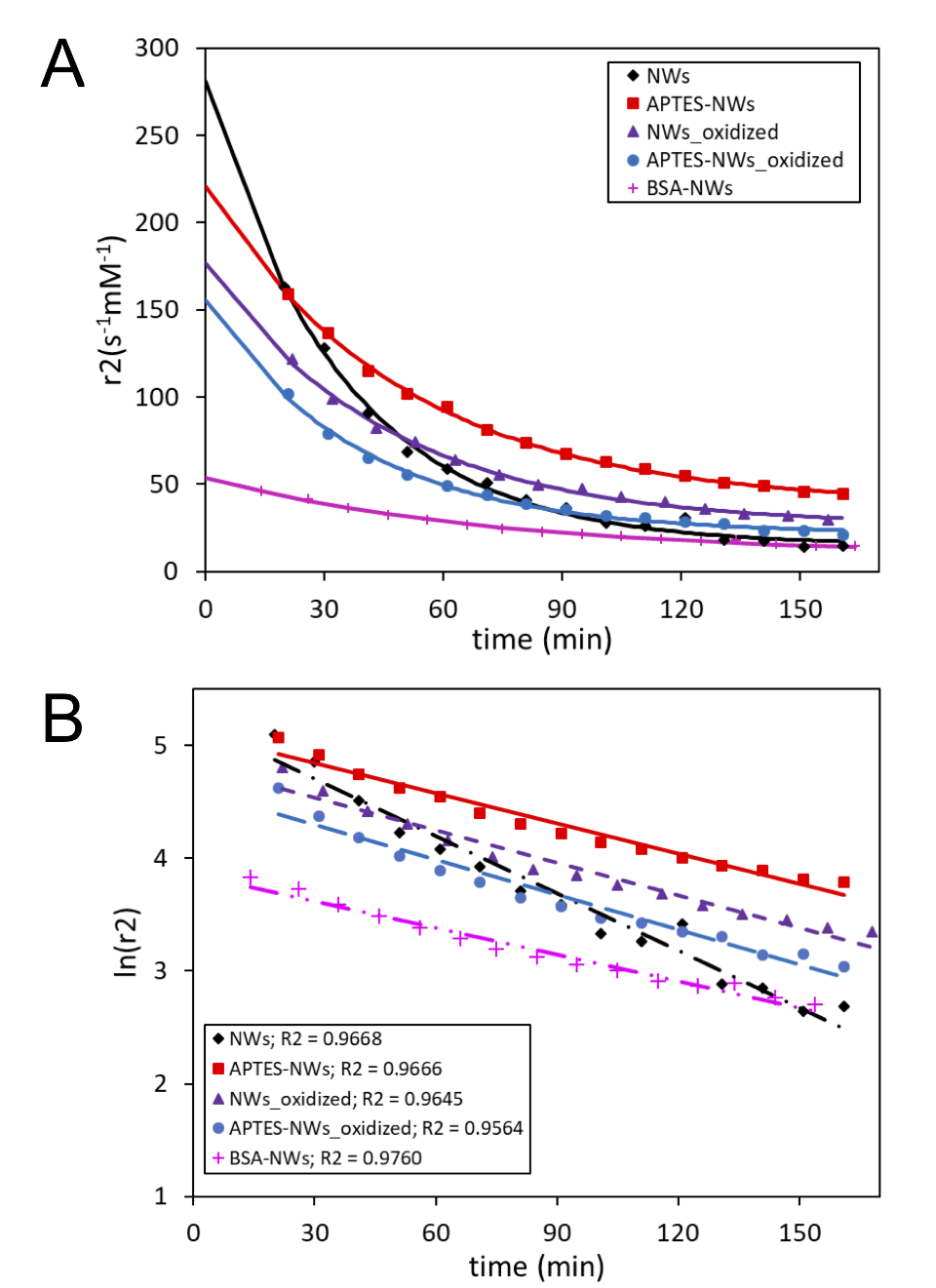
**

**Figure S3**. Decay of relaxivity (r_2_) values obtained at 7 T across the time for different NW formulations. (A) Fitting of r_2_ vs. time to a mono-exponential decay for each NW formulation from which the relaxivity at time 0 was estimated. (B) Semilogarithmic plot of the r_2_ vs. time linear fitting.


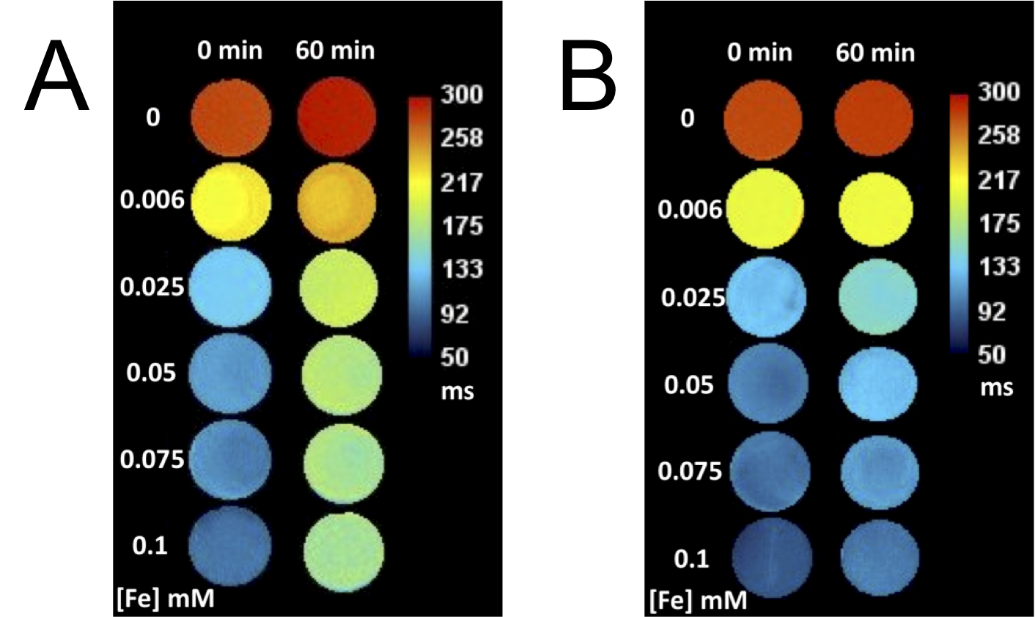


**Figure S4**. Influence of the NWs’ magnetization in the r_2_ decay across time. T2 maps of three different sets of NWs solutions. (A) The first set of NWs suspensions was measured immediately after being sonicated (A, left column) and after 60 minutes remaining all the time inside the magnet (A, right column). (B) The other two sets were sonicated at the same time with one of them being measured immediately (B, left column) whereas the second set was measured after resting for 60 min post sonication outside the magnet (B, right column).

**
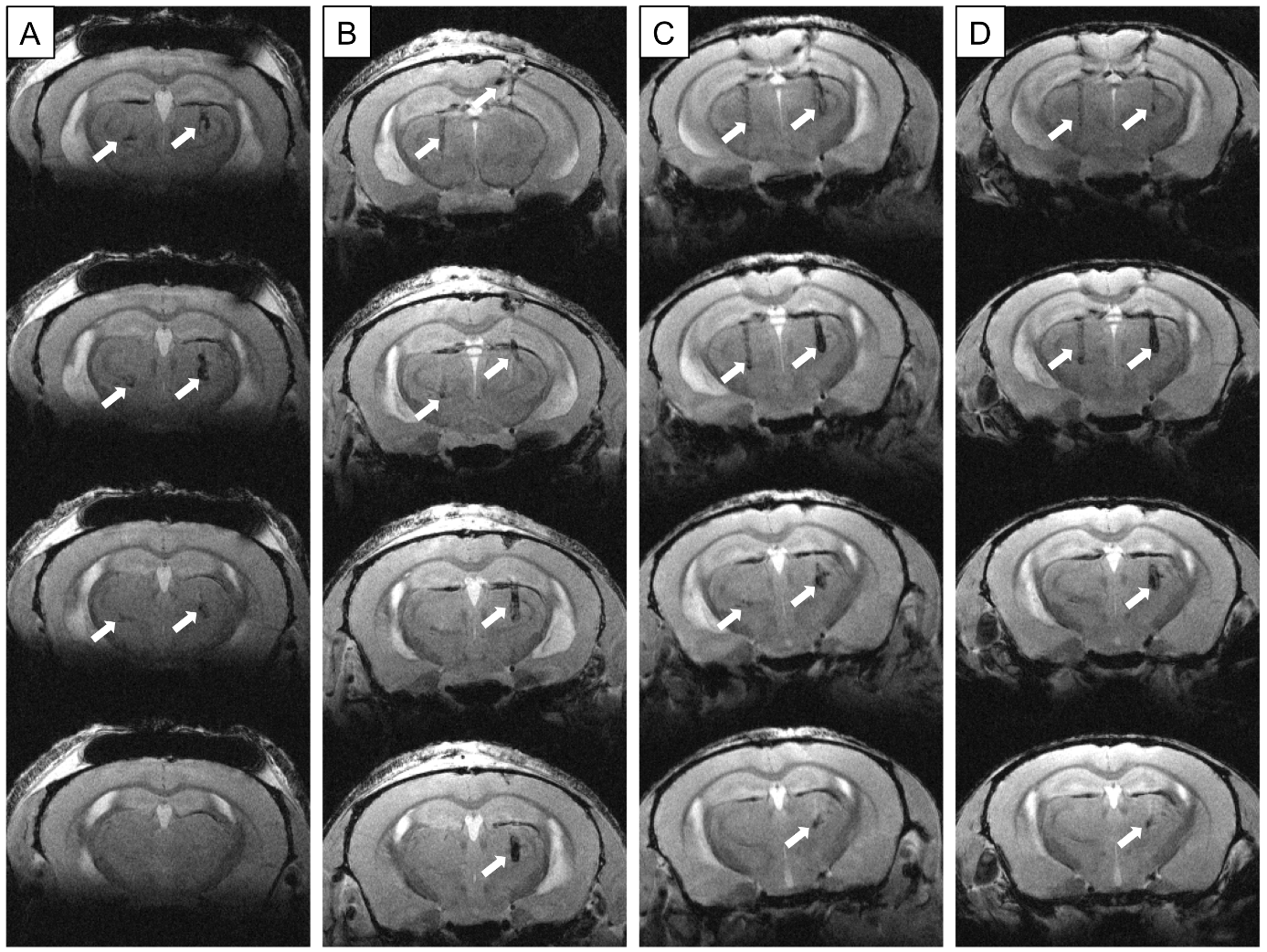
**

**Figure S5.** Four transverse consecutive slices of 200 µm of thickness across the brain of a mouse implanted with circa 10 cells labelled with BSA-NWs (left hemisphere) or circa 100 cells labelled with BSA-NWs (right hemisphere) immediately after implantation (A), and at days 10 (B), 20 (C), and 40 (D) post-implantation. White arrows point the presence of NW labeled cells.

**Additional Tables**

**Table S1**. Determination of the oxidation level of Fe NWs through elemental quantification.

| **Control Sample** | | | | **H_2_O_2_ Sample** | | | |
| --- | --- | --- | --- | --- | --- | --- | --- |
| **Relative quantification** | | | | **Relative quantification** | | | |
| Map | Element | Atomic ratio (oxygen/iron) | Element % | Map | Element | Atomic ratio (oxygen/iron) | Element % |
| 1 | oxygen | 0.25 ± 0.03 | 19.82 | 1 | oxygen | 0.34 ± 0.05 | 25.25 |
|  | iron | 1.00 ± 0.00 | 80.08 |  | iron | 1.00 ± 0.00 | 74.75 |
| 2 | oxygen | 0.26 ± 0.04 | 20.43 | 2 | oxygen | 0.50 ± 0.07 | 33.45 |
|  | iron | 1.00 ± 0.00 | 79.57 |  | iron | 1.00 ± 0.00 | 66.55 |
| 3 | oxygen | 0.15 ± 0.02 | 12.89 | 3 | oxygen | 0.74 ± 0.10 | 42.48 |
|  | iron | 1.00 ± 0.00 | 87.11 |  | iron | 1.00 ± 0.00 | 57.52 |
| Av. | oxygen |  | 17.7 ± 3.4 | Av. | oxygen |  | 33.7 ± 7.0 |
| Av. | iron |  | 82.2 ± 3.4 | Av. | iron |  | 66.3 ± 7.0 |

**Table S2**. Longitudinal (r_1_), transversal (r_2_) relaxivities and r_2_/r_1_ ratio of different nanowire formulations at 1.5 T.

| Formulation | 1.5 T  r_1_  (s^-1^mM^-1^) | 1.5 T  r_2_  (s^-1^mM^-1^) | r_2_/r_1_ |
| --- | --- | --- | --- |
| NWs | 2.2 | 70.5 | 32.0 |
| APTES-NWs | 0.6 | 38.9 | 64.8 |
| NWs_oxidized | 1.4 | 64.3 | 46.1 |
| APTES-NWs_  oxidized | 0.9 | 34.4 | 38.2 |

**Additional Methods**

**Synthesis of nanowires**

The NWs were fabricated by chemical electrodeposition into nanoporous alu­mina membranes. Briefly explaining, a 99.99 % pure aluminum substrate (Goodfellow, London, UK) was cleaned with acetone, isopropanol, and deionized water followed by an electropolishing process to even its surface. A two-step anodization of the polished alumi­num was carried out with 0.3 M oxalic acid at 4°C applying a voltage of 40V to a sealed cell containing the aluminum film and under constant stirring. This resulted in the growth of a porous anodic alumina template with hexagonally ordered nanopores with diameters from 30 to 40 nm. The first anodization process lasted for 24 h, creating inhomogeneous pores. After removal of the alumina with an aqueous solution of 0.4 M phosphoric acid and 0.2 M chromium trioxide at 40ᵒC, a second anodization lasted 4 h, yielding pores with parallel orientation and ordered arrangement. Fe NWs were grown into the alumina template by pulsed electrodeposition with current pulses limited to 60 mA employing a solution composed of 0.5 M Fe (II) sulfate heptahydrate, 0.5 M sodium sulfate, 0.4 M boric acid and 0.1g/100 mL of ascorbic acid. The NWs’ length of ~700 nm was controlled by the deposition time. Thereafter, the template containing the NWs was dis­solved with 1 M sodium hydroxide in an Eppendorf tube for 20 min, and the alumina membrane was removed. The sodium hydroxide solution was replaced every hour for four times. Suspended NWs were washed thoroughly with absolute ethanol and stored at room temperature.

**Magnetic characterization of nanowires**

50 µL aliquots of dispersed NWs with known concentrations containing either a native oxide layer or NWs subjected to oxidizing conditions were allowed to dry completely in VSM containers at room temperature. Every measurement was performed in a VSM at room temperature with a saturation field of 957.2 kA/m, a field step of 8 kA/m and averaging time of 1 second per data point. Before and after each measurement, a reference Ni sphere was measured under the same conditions to validate the consistency of the results. Measurements were performed by triplicate and expressed as average values.

**Quantification of iron nanowires**

From each formulation of NWs in aqueous solution, 100 μL were taken to a falcon tube and mixed with 300 µL of 37% hydrochloric acid. The resultant suspension was sonicated for 30 minutes at 40°C. Finally, 3 mL of bi-distilled water were added. From each of the cell suspensions used for phantom studies, 1 mL was taken and mixed with 300 µL of 37% hydrochloric acid followed by the same treatment as for the NW stock solutions. The Fe concentration was determined by measuring the samples by triplicate using an ICP-MS.

**Relaxivity measurements at 1.5 T**

Relaxivities of the different formulations of NWs were measured at 1.5 T and 37°C using a Bruker Minispec MQ60 instrument (Bruker Biospin GmbH, Ettlingen, Germany). All experiments were performed using a set of 5 serial dilutions of the contrast agent with a total volume of 300 µl per sample. The serial dilutions were prepared from a stock solution with a measured Fe concentration. T_1_ and T_2_ relaxation times of the different samples were determined using the inversion-recovery and the Carr-Purcell-Meiboon-Gill methods, respectively.^[45]^ Measurements were repeated three and five times for T_1_ and T_2_ relaxation times, respectively. Before each measurement, samples were sonicated for 20 seconds to ensure proper suspension of the NWs. T_1_ and T_2_ relaxation times were used to determine the corresponding relaxivities (r_1_ and r_2_) from the linear fitting of the relaxation rates (R_1_ = 1/T_1_ and R_2_=1/T_2_) as a function of Fe concentration in each solution (as determined by ICP-MS), using Equation 1.

**Magnetization effect of iron nanowires in the Bruker Biospec 70/30 USR scanner (7 T)** In order to determine any potential magnetization of the NWs induced by the strong equipment’s magnetic field, an experiment was planned on which three sets of solutions from a selected NW formulation were prepared, at five different Fe concentrations ranging from 0.006 to 0.1 mM. All samples were sonicated for 20 seconds before introducing them to the MRI equipment. The first set of solutions was placed into the equipment for the measurement of T_2_ relaxation times and kept inside of the equipment all the time. T_2_ relaxation times were measured just after introducing the samples in the magnet, and 60 min later. In parallel, two other identical sets of samples were sonicated, and one of them was introduced directly into the MRI equipment and measured, being considered as T_2_ for time 0, while the second set of samples remained outside of the equipment for 60 min before being introduced in the magnet and measured. r_2_ values were calculated as before, and T_2_ maps were generated for each set of samples at the chosen measurement times.

**Preparation of cell suspensions for in vitro MRI detection**

Cell suspensions were prepared from MDA-MB-231 cells labeled with APTES and BSA coated NWs. Cells were cultured on a 24-well plate at a density of 4x10^4^ cells and incubated in 500 μL of DMEM containing 10% FBS for 24 h at 37°C and 5% CO_2_ to reach confluence. Samples of APTES and BSA coated NWs were washed three times with phosphate saline buffer (PBS) solution, suspended in 2 mL of growth media at a concentration of 12 μg of Fe/mL and 20 μg of Fe/mL, respectively, and sonicated for 10 seconds for dispersion of the NWs. The growth media of four wells was removed, and 500 μL of the suspension containing each formulation of NWs were added to the cultured cells separately. In the same manner, the growth media of another four wells with cultured cells was replaced by 500 μL of fresh media to be used as a control group. After 24 h at 37°C and 5% CO_2_ the samples were washed three times with PBS to remove any free NWs. Cells were detached using trypsin-EDTA 0.25%, and both groups of cells were collected separately into falcon tubes, which were filled up with fresh growth media to a final volume of 2.5 mL. Both cell suspensions were sonicated for 10 seconds, and cells were counted by bright field microscopy with a Neubauer chamber (Superior Marienfield, Germany). Both cell suspensions were sonicated for 10 seconds, and cells were counted by bright field microscopy with a Neubauer chamber (Superior Marienfield, Germany). After this, the cell suspensions were immediately used for NW quantification and phantom studies.

**Preparation of agar gels for MRI imaging phantom studies**

Three 1.6% agar gels were prepared by dissolving 1.6 mg of low melting point agar (Agar-Agar, Sigma Aldrich) into 100 mL of miliQ water and heated to 100°C under constant steering until complete solvation. A homemade plastic mold was used for casting the gels and as a support for the imaging experiments. Thus, the agar solution was poured into the molds and left to cool down at room temperature. The gels were covered with parafilm and kept at 4°C to protect them from drying. Nine identical wells of ~5 mm diameter were drilled in each one of the gels. An agar solution of 0.9 % was prepared by dissolving 0.09 mg of agar in 10 mL of miliQ water and heated to 100°C under constant steering until complete solvation. From this solution, 30 μL were added to each well to create a flat area at the bottom of the wells and ensure perfect symmetry. Thereafter, the gels were left to cool down for 1h at room temperature.
